# Nitrogen Scavengers: History, Clinical Considerations and Future Prospects

**DOI:** 10.1002/jimd.70110

**Published:** 2025-10-30

**Authors:** Sven Klassa, Johannes Häberle

**Affiliations:** ^1^ Division of Metabolism and Children's Research Center University Children's Hospital Zurich Zurich Switzerland

## Abstract

Nitrogen scavengers play a critical role in treating acute and chronic hyperammonemia, especially in urea cycle disorders (UCDs), where impaired ammonia detoxification leads to toxic nitrogen accumulation. These agents complement low‐protein diets and urea cycle intermediates. Sodium benzoate and sodium phenylacetate are the main scavengers, conjugating with glycine and glutamine to form hippurate and phenylacetylglutamine, which are excreted in urine. This therapeutic approach, introduced in the 1980s, was based on early findings linking benzoate to reduced urea excretion. Nitrogen scavengers are also used in secondary hyperammonemia from organic acidemias and fatty acid oxidation disorders, though they may become increasingly ineffective in progressing liver failure due to their reliance on hepatocyte function. To improve tolerability, phenylbutyrate was developed as an oral alternative to phenylacetate and is available in sodium‐bound and prodrug forms, but issues with taste and side effects persist. While effective, current treatments target nitrogenous waste products rather than ammonia directly, offering an avenue of future drug development for UCDs. This review discusses the chemical properties, clinical use, and limitations of nitrogen scavengers, hereby focusing on phenylacetate and related substances, and highlights the need for improved therapies, including approaches that directly target ammonia removal. For the benefit of readers already experienced in nitrogen scavengers for UCDs, we also include considerations concerning the use of these drugs in animal experiments and a viewpoint on ornithine phenylacetate as a related substance.

## 1 Introduction

Nitrogen scavengers are one of the main therapeutic modalities to tackle acute or chronic hyperammonemia, a condition with overwhelming accumulation of nitrogenous metabolites including ammonia and glutamine. They belong, together with low‐protein diet and intermediary amino acids of the urea cycle, the liver‐situated pathway converting ammonia to urea, to the indispensable therapeutics in the management of urea cycle disorders (UCDs) [1]. Few other conditions, which are not directly involved in ammonia detoxification, impair the urea cycle function and cause secondary hyperammonemia that can also be treated with nitrogen scavengers [2]. These include organic acidemias, in which high concentrations of organic acids inhibit the critical urea cycle enzyme N‐acetylglutamate synthase (NAGS), and fatty acid oxidation disorders, which cause decreased levels of acetyl‐CoA, one of the substrates of NAGS [1, 3]. Liver failure can also cause secondary hyperammonemia, however treatment with nitrogen scavengers must take into account their mode of action requiring functioning hepatocytes to be effective even though there is early evidence pointing towards some therapeutic benefit. In this review, we will focus mainly on UCDs since they are the only group of diseases with hyperammonemia as the main hallmark of the disease, and because the other aforementioned conditions often present with different severe symptoms before hyperammonemia is of concern.

There are two principal substances that qualify as nitrogen scavengers, namely benzoate and phenylacetate, which are both coupled to sodium when applied in medical use. The scavenging of nitrogen molecules in the treatment of UCDs is achieved through different mechanisms depending on the therapeutic agent used. In the case of sodium benzoate, nitrogen is effectively captured through binding of its downstream product benzoyl‐CoA with glycine, forming hippurate, which is then excreted in the urine [4]. In a similar way, sodium phenylacetate is processed to phenylacetyl‐CoA which conjugates with glutamine to form phenylacetylglutamine, which is also excreted in the urine, effectively removing excess nitrogen from the body [5]. Both sodium benzoate and sodium phenylacetate, through these pathways, play a crucial role in managing hyperammonemia, by promoting the urinary excretion of waste nitrogen in nontoxic forms.

The first time that nitrogen scavengers have been mentioned with their capacity to treat hyperammonemia was by pioneers in UCD research Saul Brusilow and Mark Batshaw [6, 7], at that time working at the Johns Hopkins School of Medicine, who followed up on historical studies published before 1920 that showed decreased urinary urea excretion upon sodium benzoate ingestion “indicating that hippuric acid nitrogen is derived at the expense of the nitrogen normally eliminated as urea” [8]. In another study aiming to prove the existence of glutamine as a metabolite in the human organism, phenylacetylglutamine was detected in human urine after ingestion of phenylacetate [9]. Brusilow and Batshaw were inspired by these reports and successfully used these compounds in a groundbreaking attempt to treat acute hyperammonemia in a few single patients, thereby showing for the first time that this method of nitrogen scavenging is a quantitatively significant alternative mechanism of waste nitrogen disposal in patients with UCDs [10, 11]. This laid the basis for the treatment of acute and chronic, primary and secondary hyperammonemias, which is still widely practiced today.

Phenylbutyrate was introduced in 1990 with the main intention to find a compound with a less unpleasant odor and taste than that of phenylacetate [5] after the demonstration that phenylbutyrate, administered orally, is rapidly converted to phenylacetyl‐CoA in the body via beta‐oxidation. It is available in its sodium‐coupled form and its prodrug form glycerol phenylbutyrate [12, 13]. Notably, there was also some new development with changes to the drug formulations of oral phenylbutyrate [14, 15, 16], trying to improve adherence that was notoriously unsatisfactory, mainly due to the taste and the large amount and frequency of medication needed to swallow [17]. However, the targets of scavenging therapy, glycine and glutamine, remained unchanged and treatment is still not directly addressing ammonia as the main culprit of hyperammonemia.

This paper will discuss the chemical characteristics of nitrogen scavengers and their clinical use, will delineate the potential side effects of especially phenylbutyrate that may be considered for alternative therapeutic routes, and will provide an outlook for future developments including direct ammonia scavenging.

## 2 Chemical Characteristics of Sodium Benzoate, Sodium Phenylacetate and Phenylbutyrate‐Based Drugs

Sodium benzoate (CAS number 532‐32‐1, formula C_7_H_5_NaO_2_, molecular weight 144.1) exerts its therapeutic action primarily by binding to the amino acid glycine after conversion to benzoyl‐CoA [4, 5]. This binding results in the formation of hippurate, which is then excreted in the urine, helping to clear excess nitrogen from the body. Sodium benzoate is frequently used in the management of hyperammonemia due to its effective nitrogen‐scavenging properties. However, it is not a licensed pharmaceutical product but can only be purchased as a chemical compound for research purposes, which burdens doctors with challenges elaborated later in this review. The only exception to the use of the aforementioned chemical preparations is one licensed drug that contains equal amounts of benzoate and phenylacetate for intravenous use.

**FIGURE 1:**
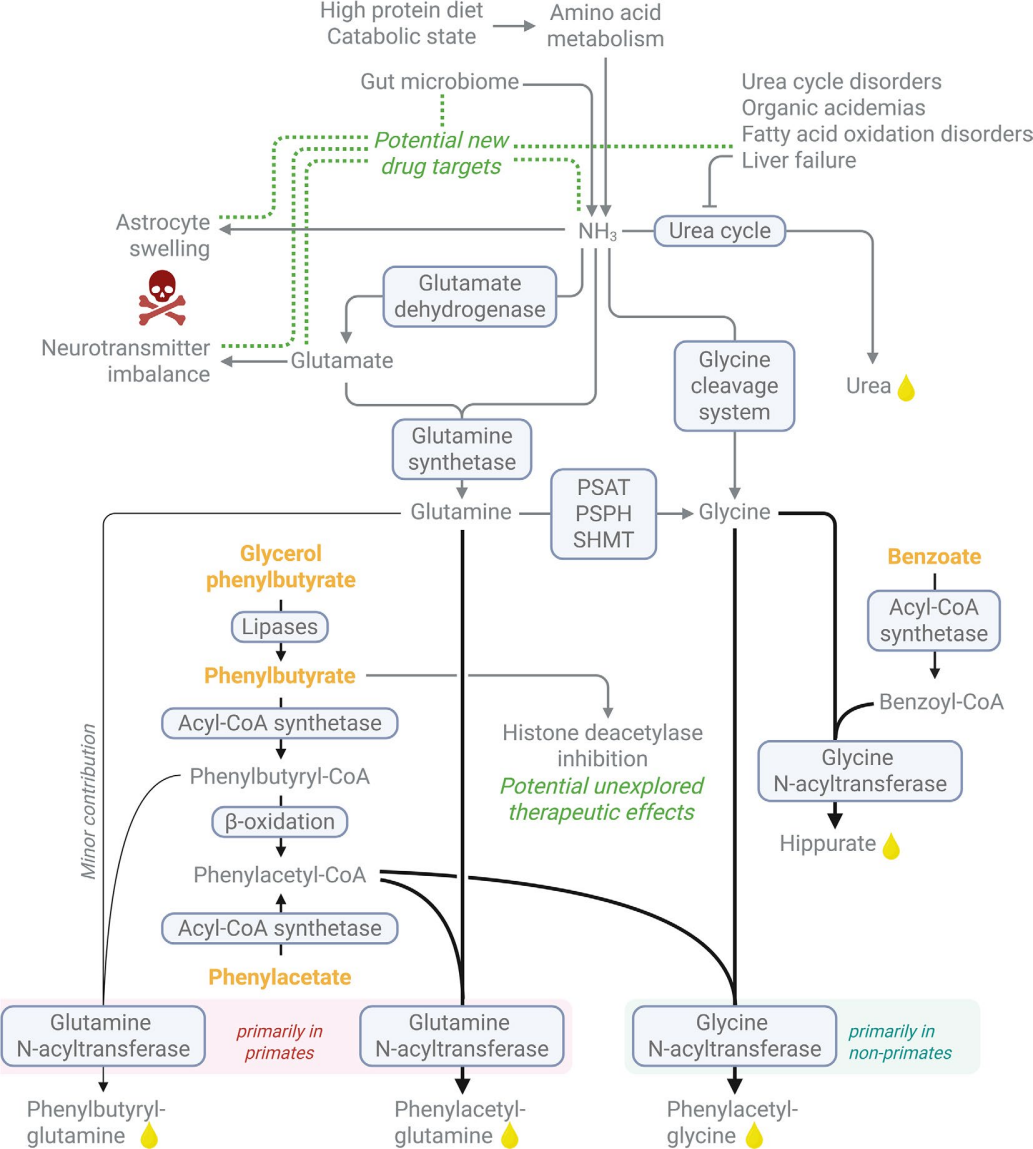
Graph depicting the fate of nitrogen scavengers (in bold orange) described in this paper as well as related pathways. Complete pathways as well as single enzymes are shown in rounded boxes with light blue background. Substances excreted in urine are shown by adding a small yellow drop of urine. PSAT: phosphoserine aminotransferase; PSHP: phosphoserine phosphatase; SHMT: serine hydroxymethyltransferase.

Sodium phenylacetate (CAS number 103‐82‐2, formula C_8_H_7_NaO_2_, molecular weight 158.1) is a another key therapeutic for hyperammonemia, but it is administered in the alternative prodrug form of phenylacetyl‐CoA, sodium phenylbutyrate (CAS number 1716‐12‐7, formula C_10_H_11_NaO_2_, molecular weight 186.2). Possibly due to the same end product it is often referred to as a prodrug of phenylacetate. However, phenylbutyrate is indeed not converted to phenylacetate but both compounds are metabolized to phenylacetyl‐CoA. One can consider phenylbutyrate a prodrug of phenylbutyryl‐CoA, which is in turn, like phenylacetate, a precursor of phenylacetyl‐CoA. Phenylbutyrate is activated in the liver to phenylbutyryl‐CoA, which could either undergo β‐oxidation to phenylacetyl‐CoA, which captures nitrogen either in the form of glutamine by forming phenylacetylglutamine or in the form of glycine to produce phenylacetylglycine, or conjugate directly to glutamine to form phenylbutyrylglutamine (Figure 1) [18]. Both products are excreted in the urine, thereby removing excess nitrogen. Sodium phenylacetate itself, like sodium benzoate, is only available as a chemical compound, except for the combination formulation with sodium benzoate.

Hippurate, phenylacetylglutamine, phenylacetylglycine and phenylbutyrylglutamine are synthetized in a two‐step pathway requiring adenosine triphosphate (ATP) and coenzyme A (CoA) to form first aryl‐CoA intermediates prior to amino acid‐specific transacylation of glycine and glutamine, respectively [19, 20].

Sodium phenylbutyrate, along with the prodrug compound, glycerol phenylbutyrate, which is cleaved by lipases to three molecules of phenylbutyrate, has been licensed for use in a variety of formulations, including oral, enteral, and intravenous forms [12, 13, 15]. It is noteworthy that to our knowledge, intravenous sodium phenylbutyrate is only used in few areas of the world, most notably in the United Kingdom [21, 22]. These formulations offer flexibility in treatment, allowing for tailored administration based on the patient's specific needs and clinical circumstances. Both versions of the drug, coupled to sodium or glycerol, are primarily used in the management of UCDs, where they play a critical role in reducing acutely elevated ammonia levels when administered intravenously, as well as in ensuring normal plasma ammonia levels long‐term when given orally.

On a molar basis, due to the presence of two nitrogen molecules in glutamine compared to the single nitrogen molecule in glycine, glutamine binding and excretion would be expected to have a higher efficacy per mole than glycine processing. This is because the two nitrogen atoms in glutamine allow phenylacetate and phenylbutyrate to potentially remove more nitrogen per molecule of the drug. However, despite this theoretical advantage, this has not fully materialized in clinical practice, probably partly because the cumulative urinary excretion of phenylacetate, phenylbutyrate, phenylacetylglutamine and phenylbutyrylglutamine amounts only to about half of the dose of phenylbutyrate suggesting the existence of additional metabolites of phenylbutyrate [18]. Research and empirical clinical experience have suggested that the relative efficacy of phenylacetate, phenylbutyrate and benzoate in nitrogen scavenging may not differ as significantly as expected, and both drugs are considered to be useful in treating UCDs and other conditions with hyperammonemia [1].

## 3 Ornithine Phenylacetate—A Viable Alternative Formulation?

We would like to mention another compound, ornithine phenylacetate which has been first suggested for treatment of hyperammonemia in hepatic encephalopathy due to liver failure [23]. The authors hypothesize that ornithine is taken up by muscle tissue and serves as the substrate to form glutamate and subsequently glutamine by incorporating an additional nitrogen molecule from ammonia through glutamine synthetase. Finally, phenylacetate binds glutamine to form phenylacetylglutamine for excretion. In many studies, ornithine phenylacetate was used in animal models of liver cirrhosis or liver failure or in patients to lower blood ammonia. Many of these studies report effective ammonia removal but clear clinical improvement could yet not be shown. The proposed mechanism of action could also be considered as a treatment for UCDs, however this compound has not yet been used in this context. We view the use of ornithine phenylacetate critical for three reasons: (1) Per molecule of ornithine, two nitrogen atoms are added to the system. The accompanying phenylacetate removes two nitrogen atoms when binding to glutamine. In total, there is a net zero change of nitrogen in the organism. Thus, a preexisting excess of nitrogen, leading to hyperammonemia, is not resolved. Merely the conversion of toxic ammonia to less toxic glutamine may take place and would explain the short term reduction of blood ammonia, however upon breakdown of glutamine and glutamate, ammonia is again released into the system, and could cause a rebound effect at a later stage. In nonprimate animal studies, in which ornithine phenylacetate has been used to lower blood ammonia [24, 25, 26, 27], the ratio seems even worse. In animals such as mice, rats and pigs, phenylacetate can only bind to glycine, not to glutamine. Hereby, only one nitrogen is excreted for two nitrogen atoms added, leading to a net 1+ nitrogen added to the system per molecule of ornithine phenylacetate administered. (2) In liver failure, as well as in UCDs, blood glutamine levels are already at normal to elevated levels since excess ammonia is by default buffered into glutamine [1, 28]. We do not see reason that boosting glutamine production by ornithine would provide a benefit in comparison to omitting it and administering phenylacetate in the sodium bound form or an alternative form without an additional amino acid increasing the nitrogen load on the organism. (3) Specifically in hepatic encephalopathy with some degree of liver damage already present, the action of phenylacetate must be hindered to some extent. The binding to glutamine to form phenylacetylglutamine is taking place in hepatocytes. If the liver is damaged so much that there are no sufficient urea cycle proficient hepatocytes anymore, it is questionable if the liver has enough capacity to form phenylacetylglutamine at clinically significant levels. In summary, we see no advantage in including ornithine in a nitrogen scavenger formulation, as we believe glutamine production does not need to be stimulated and the amount of nitrogen added matches what the scavenger can excrete.

## 4 Studies Into the Efficacy of Nitrogen Scavengers

As described above, use of nitrogen scavengers was initiated purely empirically and in a time when preclinical pharmacological and clinical bioavailability studies were not handled as stringently as nowadays. Nevertheless at present, nitrogen scavengers are the only licensed drugs for UCDs and available in different formulations to facilitate their use in acute and chronic management. Different from the early use of benzoate and phenylacetate, the recently introduced drugs underwent a rigorous licensing process including all phases of clinical trial testing [12, 13, 15, 29, 30, 31, 32].

While use of nitrogen scavengers can be considered efficacious based on the aforementioned human studies, two recent studies in dogs report no difference in reduction of hyperammonemia after treatment with sodium benzoate and/or sodium phenylacetate compared with the saline control group [33, 34]. This is in clear contrast to both empirical evidence by patients and physicians as well as the studies mentioned above clearly showing a beneficial effect from these drugs. The authors speculate that this discrepancy may be not due to ineffectiveness of the nitrogen scavengers but to the diuretic effect resulting from blood volume expansion after saline or drug injection. However, neither would this explain why the nitrogen scavengers have no additional ammonia lowering effect, nor is the injected volume specified to support this hypothesis as discussed elsewhere [35]. We propose an alternative explanation, which was not mentioned in the original publication as well as in other work citing this study. The model organism used are dogs with congenital portosystemic shunt (CPSS), an inborn defect causing a bypass of the portal vein omitting blood flow through the liver, connecting the circulation from the intestine directly to the heart and systemic circulation. As expected, this reduces the capacity of ammonia detoxification by reduced perfusion of urea cycle proficient hepatocytes, but at the same time likewise impairs the efficacy of nitrogen scavengers in the same cells. If due to the bypass the drugs and circulating metabolites do not reach the liver (or only in strongly reduced amounts), naturally these compounds will not be effective. Additionally, CPSS is generally associated with cholestasis [36], which may cause hepatocyte damage, hereby further reducing the dogs' capacity to metabolize ammonia and related compounds. As a final note, we do however want to point out that there is early evidence that nitrogen scavengers may be effective in humans with CPSS for preventing hyperammonemia [37]. Additionally, it has been shown that patients with liver cirrhosis are capable to produce phenylacetylglutamine at similar levels as healthy controls [38], provoking the question if other organs or tissues exhibit acyltransferase function for conjugation of glycine or glutamine.

## 5 The Metabolization of Phenylacetate and Phenylbutyrate Differ Strongly Between Primates and Nonprimate Species

Furthermore, the evolutionary age of enzymes involved in the metabolism of nitrogen scavengers should be considered, as the enzyme glutamine N‐acyltransferase is only present in primates while the related, evolutionarily older glycine N‐acyltransferase, is present in a plethora of species (Table 1). This is indicated already in a publication from 1972, in which the authors describe the phenomenon that primates excrete mainly phenylacetylglutamine after phenylacetate administration, while more distantly related species such as rodents excrete mainly phenylacetylglycine [20, 39]. Furthermore, a Uniprot protein‐BLAST performed with the amino acid sequence of human glutamine N‐acyltransferase (Uniprot accession Q969I3, Glycine N‐acyltransferase‐like protein 1, gene name GLYATL1) as the query sequence resulted in seven hits in mice, all with less than 40% sequence identity to the query, of which five belong to the family of glycine N‐acyltransferases or glycine N‐acyltransferase‐like proteins. All of these proteins have glycine as the substrate and none utilize glutamine in their catalyzed reactions. The absence of glutamine N‐acyltransferase in model animals such as rodents or dogs as in the above mentioned studies makes the use of phenylacetate and phenylbutyrate in animals questionable due to the noncomparability to humans. Only phenylacetylglycine can be formed through the action of glycine N‐acyltransferase for alternative nitrogen excretion, which captures only one nitrogen rather than two. In our opinion, nonprimate in vivo studies that aim to explore the action of nitrogen scavengers in humans should only be done with sodium benzoate or related compounds. Phenylacetate and phenylbutyrate can be effective to alleviate the burden of hyperammonemic animals to ensure animal welfare or to prolong survival for subsequent experiments in adult animals due to their ability to capture glycine but in this context, they are not suited as models for human biochemistry.

**TABLE 1:** Comparison of nitrogen scavengers.

Compound	Main fraction excreted in urine	Amino acid captured	Nitrogens excreted per molecule	Routes of administration
Sodium benzoate	Hippurate	Glycine	1	Oral, enteral, intravenous
Sodium phenylacetate	Primates: phenylacetyl‐glutamine Nonprimate mammals: phenylacetyl‐glycine	Primates: glutamine Nonprimate mammals: glycine	Primates: 2 Nonprimate mammals: 1	Oral, enteral, intravenous
Sodium phenylbutyrate				Oral, enteral, intravenous^a^
Glycerol phenylbutyrate			Primates: 6 Nonprimate mammals: 3	Oral, enteral

^a^ Only reported from United Kingdom.

## 6 The Properties of Phenylbutyrate Beyond Nitrogen Scavenging

Sodium phenylbutyrate is a compound with multiple mechanisms of action, hereby possibly extending its usefulness beyond the role as nitrogen scavenger in the management of UCDs.

The primary role of sodium and glycerol phenylbutyrate is the scavenging of nitrogen molecules.

In addition to its role as a nitrogen scavenger, sodium phenylbutyrate acts as a promiscuous histone deacetylase inhibitor (HDACi) [40, 41]. By inhibiting HDACs, sodium phenylbutyrate influences chromatin structure and gene expression. This action can lead to the upregulation of various genes involved in cellular stress responses, protein quality control, and apoptosis. This effect has been explored for its potential in treating a range of conditions, including cancers and neurodegenerative disorders, where dysregulated gene expression contributes to disease pathology. For example, a pathogenic variant in the Cystic Fibrosis Transmembrane Conductance Regulator (CFTR) protein causing cystic fibrosis has been considered a target for a potential therapy with sodium phenylbutyrate. The compound's HDACi action can cause an modulation of heat shock proteins like Hsp70 and Hsc70 allowing mutant CFTR protein to be processed correctly rather than being targeted for degradation, which is the root cause for disease progression [42, 43]. In a similar fashion, another study explored the potential of sodium phenylbutyrate in the prevention of the aggregation of α‐synuclein, a neuronal protein whose dysfunction is associated with Parkinson's disease, for slowing down disease progress by upregulation of molecular chaperones [44]. The drug's HDACi function may also be useful in treating sickle cell anemia by induction of fetal hemoglobin expression to compensate for deficient hemoglobin [45, 46, 47] or in cancer therapy by transforming malignant cells towards a more differentiated state [48, 49].

Beyond nitrogen scavenging and HDAC inhibition, sodium phenylbutyrate has been implicated in other biological processes. It has been shown to have antioxidant properties, potentially protecting cells from oxidative stress, which is often elevated in metabolic disorders and other chronic conditions [50]. It has also been suggested to modulate cellular signaling pathways related to inflammation and apoptosis, further contributing to its potential therapeutic effects in various diseases [51, 52].

In summary, the role of phenylbutyrate as a histone deacetylase inhibitor and its potential antioxidant and neuroprotective effects open avenues for further therapeutic applications, particularly in metabolic and neurodegenerative disorders.

## 7 Side Effects of the Nitrogen Scavengers

Sodium benzoate and sodium phenylacetate are generally considered safe medications [53, 54]. Nausea and vomiting can occur after infusion [55], and ondasteron administration is recommended for prevention [1]. Other reported adverse events can be explained by the underlying UCD in most cases, like seizures, brain edema, and hyperammonemia. Hypokalemia can be a side effect due to the diuretic effect of the compounds, causing electrolyte imbalance [56]. The odor of phenylacetate is universally described as strong, unpleasant and persistent [57], which needs to be considered when preparing the administration to ensure minimal exposure to avoid discomfort of patients and medical staff. Deaths have been reported in patients that received an overdose but it is possible that the underlying cause is a life threatening metabolic decompensation caused by the UCD while high doses of nitrogen scavenger are only a confounding factor. A study reporting on three UCD patients receiving inappropriately high doses of sodium benzoate and sodium phenylacetate describe agitation and confusion, notwithstanding decreased plasma ammonia, metabolic acidosis and depressed mental status. Two patients died with cerebral edema and one patient survived after hemodialysis [56].

In 2020, a study reported an association between blood phenylacetylglutamine, derived from gut microbial phenylacetate, and cardiovascular disease, mediated by a direct activation of platelet adrenergic receptors [58]. Previously, in 2016, a prospective study of patients with chronic kidney disease showed a correlation of serum phenylacetylglutamine with cardiovascular disease risk [59]. Since then, in recent years multiple studies have reported similar findings [60, 61, 62], however without pointing out the fact that UCD patients receive phenylacetate and ‐butyrate formulations that cause much higher phenylacetylglutamine levels (mean plasma c_max_ of up to 460 μM with slow elimination [29, 63]) than those produced from microbial phenylacetate (mean plasma concentration below 10 μM [58, 61, 62]). So far, it has not been reported that UCD patients receiving nitrogen scavengers are at a higher risk of cardiovascular disease, which is why we do not recommend to abstain from phenylacetate based nitrogen scavengers [64, 65, 66]. However, there are isolated incidents of cardiac disease reported in patients receiving phenylacetate and benzoate [54] but a potential association has not been quantitively examined in detail and there is no recommendation to specifically monitor cardiac function in clinical practice. Further studies are needed to investigate why microbially derived but seemingly not pharmaceutically derived phenylacetylglutamine are associated with cardiovascular disease.

Sodium and glycerol phenylbutyrate may also cause a range of side effects, but are usually limited to gastrointestinal distress with nausea, vomiting, diarrhea, and abdominal pain [29, 30, 67, 68]. These symptoms are often dose‐dependent and may occur if high doses are required for treatment. Some patients may experience tiredness, fatigue, or drowsiness, as well as loss of appetite, which may lead to unintended weight loss, possibly even leading to a vicious circle perpetuating hyperammonemia. There may also be effects on blood cells, including leukocytopenia and thrombocytopenia, although these are less common and typically occur in patients younger than 2 years. Allergic responses, including skin rashes, itching, or more severe hypersensitivity reactions, can occur but are rare.

Besides these aforementioned adverse events, it has been observed that phenylbutyrate, particularly when used over long periods, may have an impact on ovarian function. Approximately one quarter of fertile female patients reported irregular periods or a complete abstinence of their menstruation [69, 70]. The exact mechanisms by which this could occur are not fully understood. As a HDAC inhibitor, sodium phenylbutyrate has the ability to alter gene expression, which could theoretically affect reproductive hormones and ovarian function. More research into understanding the pathogenesis of this adverse effect is needed, also to evaluate long term risks on reproductive health and the endocrine system.

## 8 Current Clinical Situation Regarding Use of Nitrogen Scavengers

Sodium benzoate and sodium phenylacetate are available in both oral and intravenous formulations, yet neither of these have been officially licensed as a drug. Instead, they are classified as chemicals only for research purposes, meaning they are not subject to the same regulatory oversight as licensed pharmaceutical products. As already mentioned above, a combination of benzoate and phenylacetate, each at 10% concentrations, exists as a licensed drug. Furthermore, it is practice in some metabolic centers to concomitantly use the chemical preparations of both compounds for intravenous application, which, according to anecdotal evidence, is likewise effective. In the given situation, healthcare providers must offer specific information when administering sodium benzoate or sodium phenylacetate to patients in clinical practice. Despite its widespread and longstanding use, especially in the management of UCDs, the history of sodium benzoate's introduction has largely been empirical, based on its effectiveness observed in clinical settings rather than through formal drug development processes. This lack of formal licensing has, unfortunately, not prompted the pharmaceutical industry to develop a licensed formulation of sodium benzoate. As a consequence, healthcare providers and patients continue to rely on unlicensed chemical forms, which may lead to inconsistencies in quality control, dosing, and patient safety. The absence of a licensed, commercially available product has remained a significant gap in the therapeutic options for UCDs, even though sodium benzoate has proven to be a valuable tool in managing ammonia toxicity.

The situation is quite different for the other compounds. Sodium and glycerol phenylbutyrate are licensed as pharmaceutical drugs for oral administration in large parts of the world, both of which are approved for use in the treatment of UCDs [12, 13, 29]. Sodium phenylacetate, due to its strong odor, is only available as a drug for injection. Several licensed formulations have undergone rigorous regulatory approval, ensuring consistency in quality, safety, and efficacy. They provide flexibility in treatment, catering to different patient needs, and are considered a standard, clinically validated approach to managing ammonia toxicity. The availability of phenylbutyrate‐based formulations in licensed, regulated forms marks a significant advancement in UCD therapy compared to sodium phenylacetate, ensuring both healthcare providers and patients can rely on the drug for safe and effective management with satisfactory compliance [71].

## 9 Alternative Ammonia Scavengers and Other Future Routes

Are there alternatives to traditional nitrogen scavenging therapies in the treatment of hyperammonemia? This question opens the door to exploring novel approaches that go beyond the conventional nitrogen‐scavenging drugs [72]. While these therapies have proven effective in managing ammonia levels, they are indirect in that they do not directly capture ammonia and may not address the full spectrum of the disorder's toxic effects, particularly the neurotoxic impact of elevated ammonia levels on the central nervous system. Given that ammonia is the primary neurotoxic culprit in most of the ureagenesis defects, a more targeted therapeutic approach could potentially offer improved outcomes.

One possibility is the design and development of substances that specifically target ammonia itself rather than glycine or glutamine. For example, compounds could be developed that target ammonia to efficiently neutralize or convert it into less toxic forms before it even has a chance to affect the brain. This can also include approaches like peritoneal dialysis via injected stable liposomes capturing ammonia by ionizing the molecule on the inside, preventing its release. These liposomes are then removed from the peritoneal space [73, 74, 75, 76]. Similarly, compounds or other solutions to decrease glutamate accumulation in the brain, causing neurotransmitter imbalance, can be explored as a more upstream target for therapy.

Additionally, research could explore the development of compounds that protect the nervous system from the harmful effects of ammonia. These might include neuroprotective agents that reduce the ability of ammonia and glutamate to interfere with neurotransmission or prevent its accumulation in the brain. As well, targeting ammonia production in the intestine and its microbiome (especially urease expressing microbes, converting urea to ammonia and carbonic acid) and uptake therefrom could serve as an alternative to address ammonia toxicity, and this approach was already tested preclinically [77, 78]. Finally, genetic therapies aimed at restoring or compensating for deficient enzymes in the urea cycle could represent a promising long‐term solution [79, 80, 81], potentially eliminating the need for continuous nitrogen scavenging altogether.

In summary, while nitrogen scavenging remains a cornerstone of UCD management, in addition to low‐protein diets and supplementation of arginine and citrulline, there is significant potential for developing more targeted approaches that address ammonia as the primary neurotoxic agent. By developing new substances that either neutralize ammonia or protect the nervous system from its harmful effects, more effective and specialized therapies for patients at risk for hyperammonemia will hopefully become available.

## Conflicts of Interest

The authors declare no conflicts of interest.

## Data Availability

The data that support the findings of this study are available from the corresponding author upon reasonable request.
